# A method for interoperable knowledge-based data quality assessment

**DOI:** 10.1186/s12911-021-01458-1

**Published:** 2021-03-09

**Authors:** Erik Tute, Irina Scheffner, Michael Marschollek

**Affiliations:** 1 Peter L. Reichertz Institute for Medical Informatics of TU Braunschweig and Hannover Medical School, Hannover Medical School, Carl-Neuberg-Str. 1, 30625 Hannover, Germany; 2 Department of Nephrology, Hannover Medical School, Hannover, Germany

**Keywords:** Information science, Data quality, Data aggregation, Health information interoperability, Knowledge bases

## Abstract

**Background:**

Assessing the quality of healthcare data is a complex task including the selection of suitable measurement methods (MM) and adequately assessing their results.

**Objectives:**

To present an interoperable data quality (DQ) assessment method that formalizes MMs based on standardized data definitions and intends to support collaborative governance of DQ-assessment knowledge, e.g. which MMs to apply and how to assess their results in different situations.

**Methods:**

We describe and explain central concepts of our method using the example of its first real world application in a study on predictive biomarkers for rejection and other injuries of kidney transplants. We applied our open source tool—openCQA—that implements our method utilizing the openEHR specifications. Means to support collaborative governance of DQ-assessment knowledge are the version-control system git and openEHR clinical information models.

**Results:**

Applying the method on the study’s dataset showed satisfactory practicability of the described concepts and produced useful results for DQ-assessment.

**Conclusions:**

The main contribution of our work is to provide applicable concepts and a tested exemplary open source implementation for interoperable and knowledge-based DQ-assessment in healthcare that considers the need for flexible task and domain specific requirements.

**Supplementary Information:**

The online version contains supplementary material available at 10.1186/s12911-021-01458-1.

## Background

Planned multiple use of electronic patient data as well as reuse not anticipated at the time of data capture, e.g. for medical research, are often mentioned promises of Medical Informatics [1, 2]. Many technical and organizational challenges have to be solved, keeping it a current research topic [3, 4]. Data quality and lack of knowledge about datasets are common challenges for reuse mentioned in the literature. In this context, data quality denotes the ability of data to “serve the needs of a given user pursuing specific goals” [5]. Although there has been a consensus for many years that DQ is important and many DQ-assessment methods have been proposed, established reporting standards defining compilations of MMs for different DQ-assessment situations are still missing [5–10]. A MM is a specification of a method that quantifies a characteristic of a dataset (cf. [11]). Characteristics often examined in DQ-assessments are completeness and correctness of the dataset (cf. [5]). MMs calculating absolute and relative counts per variable, per value in this variable (absolute and relative frequencies) or counted for a certain dimension, e.g. number of values in a variable per patient, can give hints on completeness [12]. Checking constraints for valid variable’s values, e.g. range or format expectations, can indicate correctness-issues. MMs describing the distribution of values, e.g. mean and standard deviation, or extreme values like minimum and maximum, could also indicate implausible data. Reporting standards, i.e. compilations of MMs, and tangible knowledge on which results indicate ‘good’ or ‘bad’ DQ are what we refer to as DQ-assessment knowledge. Reasons for a lack of DQ-assessment knowledge discussed in the literature include general underreporting of DQ-assessment steps and a lack of comparability between MMs. For the purpose of DQ-assessment in comparative effectiveness and patient centered outcomes research, Kahn et al. [13] proposed a set of DQ relevant characteristics to be reported about a dataset based on years of experience in major research networks. However, these recommendations are not specific enough to ensure comparability if implemented independently. Furthermore, which MMs provide sensible information and assessment of their results may depend on the planned data usage [14] and the role of the person assessing the DQ [8, 15–19]. Stausberg et al. [20] suggest in their review that research should take into account proposals for formal definitions of DQ-indicators as well as standards for data definitions. Formal definitions of DQ-indicators (equate MMs) that are decoupled from the software that applies them is what we refer to as the knowledge-based approach in this paper. Changes to MMs governed separately from the applying software do not require changes to the software’s source code, thus shifting the ability for MM-governance away from software developers towards domain experts. In a recent work on a systematic DQ-assessment process Diaz-Garelli et al. [21] stress that adapting DQ-assessment to the task at hand is important, but defining DQ-requirements is a complex and resource-intensive task, typically requiring a multidisciplinary team. Governance of MMs in a knowledge-based approach could support this multidisciplinary collaboration. Furthermore, knowledge-based MMs are easier to reuse and share in different technical and organizational contexts. Two popular open source tools for DQ-assessment on health data are Achilles Heel [6] and the PEDSnet Data-Quality-Analysis [8, 19]. Both rely on the OMOP data model and implement DQ-assessment knowledge directly, without a knowledge-based approach. In epidemiological research, existing implementations of generic methods exist as R-based implementations [22–25]. R is a programming language for statistical computing. Kapsner et al. [18] implemented their DQ-framework as R-functions and mention plans to support their framework with an ISO/IEC 11179 metadata repository. Juarez et al. [26] recently published work based on such a metadata repository, in which simple constraints are stored centrally together with metadata like variable definitions. Utilizing standards like ISO/IEC 11179 for data definitions as proposed by Stausberg et al. is one aspect of interoperability. Juarez’s storage approach for constraints is a simple knowledge-based approach, but is limited to constraint checks for single variables and does not address task and domain dependency. Johnson et al. propose formally defined DQ-indicators and argue for the need to consider domain and task dependency in DQ-assessment [11, 14, 27]. Domain refers to the clinical content the data represents and its context. Task refers to the purpose of the DQ-assessment. A knowledge-based approach to DQ-assessment that considers task and domain specific requirements, that flexibly supports any kind of MM and adds means to address interoperability could help to reach well-defined, collaboratively governed DQ-assessment knowledge for different purposes in the context of healthcare.

## Objectives

The aim of this paper is to present our method for interoperable, knowledge-based DQ-assessment and findings from its first real world application. Interoperable implies two things: First, portability on standard-compliant technical infrastructure; Second, MMs base on standardized data definitions and MM-results remain comparable as long as the same or similarly structured data definitions are used. Knowledge-based implies that the MMs themselves, which MMs are applied as well as assessment of MM-results, can be expressed in a formalized way. This intends to support the long-term vision of collaboratively governing DQ-assessment knowledge considering domain and task specific requirements.

## Methods

### Technical setting

As part of the German Medical Informatics Initiative [28, 29] the HiGHmed consortium aims to facilitate meaningful reuse of data by establishing a shared information governance framework, data integration centers and an open platform architecture that makes use of several interoperability standards [30]. Out of these standards, the openEHR specification [31] provides four features helping to reach the objectives of this work. First, the specification provides definitions for basic building blocks from which complex clinical information models (CIM) can be built. Some of these basic definitions, the reference model types, can be used to automatically generate MMs similar to MMs based on a datatype, e.g. measures of distribution for numeric variables or frequencies for string variables. Second, CIMs provide shared machine-readable definitions of the clinical concepts the data represents. CIMs in openEHR are called archetypes or templates, depending on their purpose. While archetypes define a clinical concept, e.g. blood pressure, regardless of the use case, templates assemble and constrain archetypes considering a specific use case. Source systems providing data from different units or sites work in different clinical processes. Thus, the actual data instances, called compositions, usually conform to different templates. Yet, the archetypes for the same clinical concepts are still common, enabling tools to work on data from different sources through archetype-paths. The archetype-paths unambiguously reference variables within MMs in a standardized way. By this, MMs can quantify aspects of DQ for datasets from different sources in a comparable manner. Beyond that, CIMs can express constraints on valid data instances for variables, such as ranges, formats, value sets, datatypes, cardinalities. Based on this information MMs checking these constraints can be generated. Existing tools [32] and established governance processes (e.g. [33, 34]) for openEHR CIMs can support the collaborative definition and governance for such type of MM. The openEHR REST API specification for data repositories (third openEHR feature used) defines interfaces which applications can use to interact with the repository, e.g. for standardized data retrieval, by that enabling portability of tools between compliant data repositories. The Archetype Query Language (AQL) [35] (fourth used openEHR feature) is a query language similar to the well-known structured query language (SQL). AQL allows flexible querying of the data repository on the basis of CIMs, i.e. based on archetype-paths, and hence independent of local database schemata. The combination of REST API specification and AQL enables standardized, clearly defined and flexible data retrieval.

**Fig. 1:**
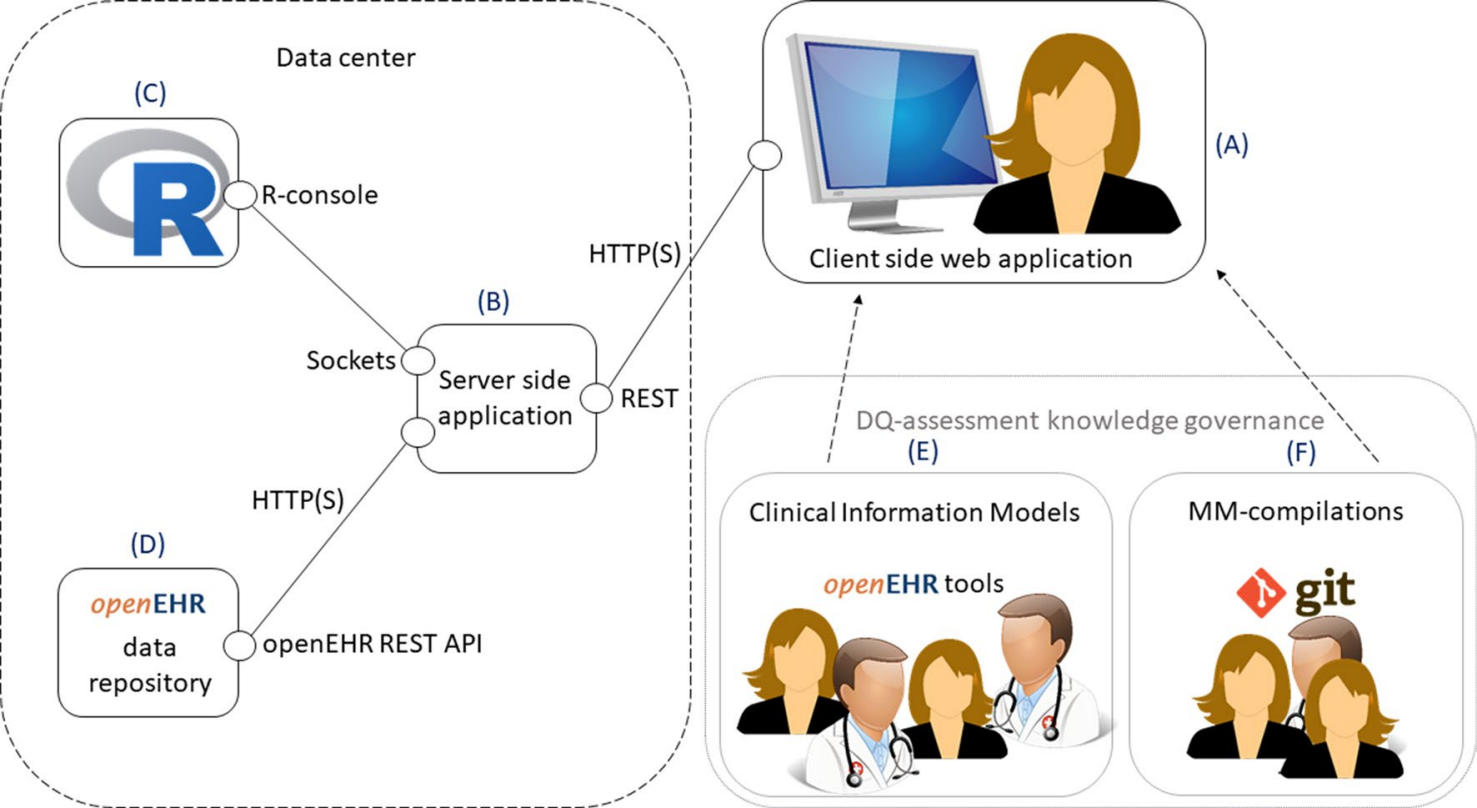
openCQA architecture overview. **A** Client-side web application, **B** server side application, **C **R for statistical computing, **D** openEHR REST API compliant data repository, **E** CIM based knowledge governance, **F** MM-compilation based knowledge governance

We implemented an open source DQ-assessment tool named openCQA [36] that makes use of the openEHR features described above. This tool implements the concepts for interoperable, knowledge-based DQ-assessment presented in this work. It consists of a web application for user interaction on client side (Fig. 1A) and a server side application for data processing implemented as Node.js application (Fig. 1B). Thus, data can stay in the institutions data center reducing potential performance and security issues. Further, openCQA makes use of a server side instance of R for statistical computing (Fig. 1C) and requires an openEHR REST API compliant data source (Fig. 1D). Two means for DQ-assessment knowledge governance are available: First, we take advantage of tools [32] and processes (e.g. [33, 34]) for openEHR CIM governance to govern constraints on valid data instances expressed in CIMs (Fig. 1E). openCQA can automatically derive applicable MMs from openEHR CIMs to check these constraints. Second, we use the version control system git [37] to manage knowledge bases, i.e. compilations of MMs for certain domains and/or tasks (Fig. 1F). openCQA can import and apply such knowledge bases as well as export MM-compilations into a knowledge base. A working instance of openCQA was available at the medical data integration center of Hannover Medical School.

### MM formalization

**Fig. 2:**
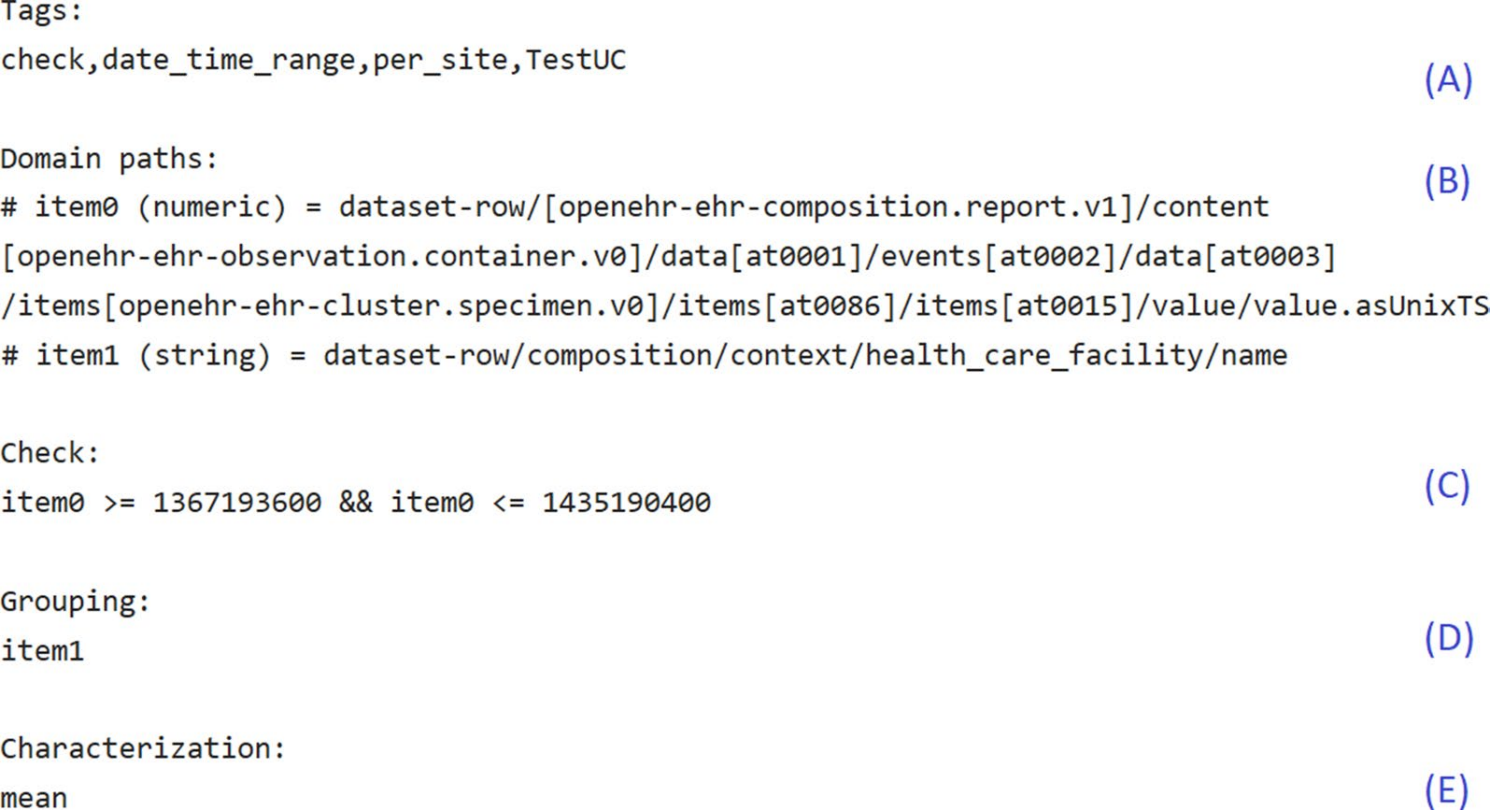
Example MM as 5-tuple checking an arbitrary range constraint on a time variable returning the mean compliance to a range constraint per clinical site. **A** Tags describing the MMs purpose and if applicable domain and task, **B** domain paths, **C** optional constraint checking, **D** optional grouping, **E** characterization function

We define MMs as simple 5-tuples as depicted in Fig. 2.

As first tuple element, *tags* are descriptive keywords indicating what the MM does and optionally the context for its intended use (Fig. 2A).

Second tuple element, is the definition of the input data for the MM by means of *domain paths* (Fig. 2B). A MM can require input data in multiple vectors. The item* in the domain path is the name of the input variable for the MM followed by the R-datatype expected for this variable. A domain path can relate to a variable by specifying where to retrieve the appropriate data from the dataset. We employ openEHR archetype-paths for that enabling us to address identical variables retrieved from different templates using the same domain path. The second type of domain path relates to other MMs, i.e. by specifying a filter-expression defining the MMs and which attributes of the MMs shall constitute the input data for this domain path. The second type of domain path enables multi-level MMs, e.g. for using results from MMs as input for another MM. Table 1 lists examples for possible types of domain paths.

**Table 1:** Examples for domain paths

Domain path example	Comment
# item1 (string) = dataset-row/composition/ context/health_care_facility/name	Constant *dataset-row* followed by an openEHR archetype-path specifies which variable from the dataset constitutes the input for item1
# item0 (numeric) = dataset-row/[openehr-ehr-composition.report.v1]/content[openehr-ehrobservation.laboratory_test_result.v1]/data [at0001]/events[at0002]/data[at0003]/items [at0098,'biopsy result final'] /value.countChildnodes(only_child)	The archetype-path is followed by an instruction.*.countChildnodes(only_child)* indicates that not the contents in this archetype-path are of interest but the number of their child nodes
# item0 (numeric) = other_data_input: (iMM.tags.indexOf("check")>-1) && (iMM.tags.indexOf("per_")==-1).resultsValue	The domain path retrieves its input data from other MMs complying with the filter *(iMM.tags.indexOf("check")*>*-1) && (iMM.tags.indexOf("per_")*==*-1)*. The instruction*.resultsValue* defines that the MM-results constitute the input data

Third part of a MM is the optional *constraint checking* (Fig. 2C). A rule is applied to each row of the input data. The rule is expressed in R. This results in a vector containing the check’s results, which is typically Boolean but yet, is not restricted to this datatype, e.g. a numeric scale would be a possible result. Table 2 lists examples for constraint checks.

**Table 2:** Examples for constraint checks

Check example	Comment
item0 >= 1 && item0 <= 1	Cardinality check for a mandatory variable. (The domain path for item0 will specify that the number of child nodes of the element at the given archetype-path is of interest, similarly to the example in Table 1.)
is.element(item0, c("Weiblich","Männlich","Divers"))	Checks if value in item0 is in list of allowed values
if (item1 == "kg") {return (item0 >= 0.0 && item0 <= 1000.0)}if (item1 == "g") {return (item0 >= 0.0 && item0 <= 1000000.0)}	Checks range constraint for valid numeric values in item0 considering the corresponding value for the unit in item1

The optional *grouping* rule (Fig. 2D) results in a vector assigning each row to a group. For example, if the number of range-constraint violations in a study’s dataset is of interest separated for each hospital, this rule defines how to group the dataset rows in this dimension, i.e. to group rows depending on the value of variable healthcare facility (cf. Fig. 2). The MM-result will contain one value per site, indicating the number of violations for each site. If no grouping is specified, all rows constitute one group. Grouping rules are expressed in R. Table 3 lists examples for groupings.

**Table 3:** Examples for groupings

Grouping example	Comment
item1	Group by value in item1. Depending on the domain path this can be used to group per site (cf. Fig. 2), time interval (year, month, quarter, day), day of week, patient etc
sprintf("%s_%s",item1,item2)	Combines multiple values for grouping, e.g. to get counts of antibiotic resistant isolates per bacteria species in lab values

The last MM-part is the *characterization* function (Fig. 2E) producing the desired MM-results. Here, characterization denotes a simple procedure summarizing a certain characteristic of a given dataset to make the contained information graspable by reducing irrelevant information. For example, in most cases, it is not of interest which item of a vector contains which value, but the overall distribution is of interest and can be expressed with measures like mean and variance or as histogram. The characterization function is a freely programmable R-function. It is not limited to predefined R-functions. Thus, the possibilities of desired output are manifold, including visualizations. Table 4 list examples for characterization functions.

**Table 4:** Examples for characterizations

Characterization example	Comment
function(v) {sum(!is.na(v))}	Count present values in variable
function(v) {mean(abs(diff(v)), na.rm = TRUE)}	Mean density of values in variable
function(v) {#begin_plot par(las = 2) par(mar = c(7,4,1,1)) barplot(v, main = NULL, xlab = NULL, names = item1, col = rainbow(length(v))) #end_plot}	Plotting a barplot for variable’s values (Before execution #begin_plot and #end_plot markers are replaced with R-code to integrate the resulting plot with openCQA)

Executable parts of the MMs are expressed in R (Fig. 2C–E). Other means considered for expressing these parts of the MMs were Drools [38], Arden Syntax [39], Object Constraint Language [40] and the openEHR Guideline Definition Language [41]. After numerous discussion with colleagues of different backgrounds and affiliations, we chose to use R. The advantages of R are manifold existing statistical methods and its popularity in some potential user groups, e.g. epidemiologists. We operationalized the 5-tuples utilizing a generic R-script as template. Additional file 1: Appendix A provides an example of an operationalized MM. Only the five attributes annotated with A–E in Fig. 2 differ between MMs.

The segmentation of parts C, D and E as well as using multi-layered MMs instead of expressing everything in one script intends to improve comparability. When mixing characterization (e.g. mean for a variable’s values) with rule checking (e.g. is value in permissible range), grouping (e.g. per hospital), adding some aggregation (e.g. a mean over some MM-results with different weights) and a visualization or assessment (e.g. dataset is OK), much variability between two MMs is introduced, since each step can slightly differ. As a result, even MMs quantifying almost similar aspects of DQ may differ in minor details, and results may not contain all necessary information from the dataset to make their results comparable. Separating these steps and defining preferably plain MMs aims to maintain comparability as long as possible.

### Application of DQ-assessment method

Example use case is the ROCKET study [42] on predictive biomarkers for rejection and other injuries of kidney transplants. We already integrated the study’s dataset into an openEHR data repository at Hannover Medical School for further analysis, dissemination and later reuse. To validate the data integration pipeline, we already compared the original data export from an electronic data capture system and the dataset in the repository. These first two steps, did not involve the methods presented in this manuscript. openCQA was developed using dummy-data and a local test instance of an openEHR data repository. The ROCKET study was the first real world application including the roll out at the data integration center. This entailed dealing with another repository as data source (same product [43] but different version) and a new domain, i.e. other CIMs. Note, that no fitting of the tool to this particular domain was required, since the method is applicable to any compliant data source with any AQL-query due to the use of standardized means for data retrieval and MM generation (cf. Technical setting). One intention of this application was to test if our described theoretic concepts for interoperable and knowledge-based DQ-assessment work, e.g. regarding portability and whether the generated MMs provide useful and correct results. For the latter purpose, one of the authors (IS) created and applied basic statistics using IBM SPSS Statistics 25 (a statistics software package) for an agreed on subset of the data-export from the electronic data capture system. These included frequencies and percent values for the nominal or ordinal data (e.g. diagnosis) and summary measures for scale variables. Another author (ET) independently derived MMs for basic statistics and computed results by applying openCQA on the data in the openEHR data repository.

As recommended for systematic DQ-assessment in the literature [21], DQ-assessment with openCQA started by precisely specifying the information of interest. The client part of openCQA (Fig. 1A) allows to do that in a standardized and executable form as AQL queries. We specified seven AQL queries each retrieving the data of interest for specific questions of our DQ-assessment. Additional file 2: Appendix B shows an example query. The client forwarded the AQL to the server side application (Fig. 1B) which retrieved the data via REST API from the repository (Fig. 1D) and sent the archetype-paths occurring in the dataset along with their respective reference model types to the client (similar to the information which variables exist in the dataset and their respective datatypes).

Our next step using openCQA was to define information needs about the dataset, e.g. DQ-requirements the dataset should fulfill (cf. [21]) or visual methods for DQ-assessment, which are common practice [22, 44–46]. A common problem mentioned in the literature is that domain experts are often left alone with this complex and resource intensive task, ending up in single-use project-specific MMs [21, 22]. Two of our concepts address this: First, automatic generation of MMs depending on the variable’s reference model type and second, the knowledge-based approach. We used the openCQA client (Fig. 1A) to derive MMs based on the reference model types calculating simple characterizations, e.g. mean value for scale variables or frequencies for categorical data, and simple visualizations, e.g. a barplot.

To keep the set of openCQA’s MMs concise when comparing generated MMs to statistics calculated in SPSS, we removed all MMs including dimensions, e.g. MMs calculating additional measures grouped per hospital. We checked if all measures of interest for our assessment were present. The measures calculated by IS in SPSS defined which measures were of interest and ET checked if these were present in openCQA’s generated MMs.

We extended openCQA’s MMs with histograms showing distributions of age for kidney transplant recipients and organ donors as well as distribution of time in months between the transplantation and the patient’s posttransplant evaluation visit that included a transplant biopsy.

As last information need, we adapted and implemented three MMs proposed by Johnson et al. for their HDQF DQ-assessment framework [11, 14, 27] and expressed them as applicable MMs in openCQA (Additional file 3: Appendix C details the adaptions to HDQF’s MMs; The MMs from HDQF framework had no counterpart in SPSS.) (1) *Representation Complete* calculates the percentage of non-missing entries per variable. (2) *Task complete* quantifies the number of missing values in mandatory variables. (3) *TDConstraints* summarizes the checks of constraints for variables defined in given CIMs.

CIMs can serve to express both domain and task-dependent constraints. A regular CIM used for data processing should already include sensible constraints, e.g. the height archetype could constrain, that the height of a person should not exceed 300 cm or a template could define mandatory context variables according to local clinical processes. Since openEHR repositories enforce compliance of data with these constraints, checking them in DQ-assessment would be pointless for data queried from an openEHR repository. However, not all suspicious values are actually wrong data. This is why constraints in regular CIMs should be set with caution to prevent excluding unusual but correct data (cf. Table 2 in [47]). In contrast, DQ-assessment wants to detect suspicious values, and therefore CIMs defining constraints for DQ-assessment need to be more extensive and restrictive (cf. Richter et al. [48]—Table 1—plausibility and admissibility limits). In addition to that, CIMs could define task dependent constraints, e.g. when a multi-disciplinary project team collaboratively decides to make fields mandatory, considering a certain planned analysis (cf. [21]). The presented method can deal with an unlimited number of CIMs (archetypes and templates) for the same clinical concept. This allows users to add CIM-based constraints arising from different roles and perspectives in DQ-assessment.

**Fig. 3:**
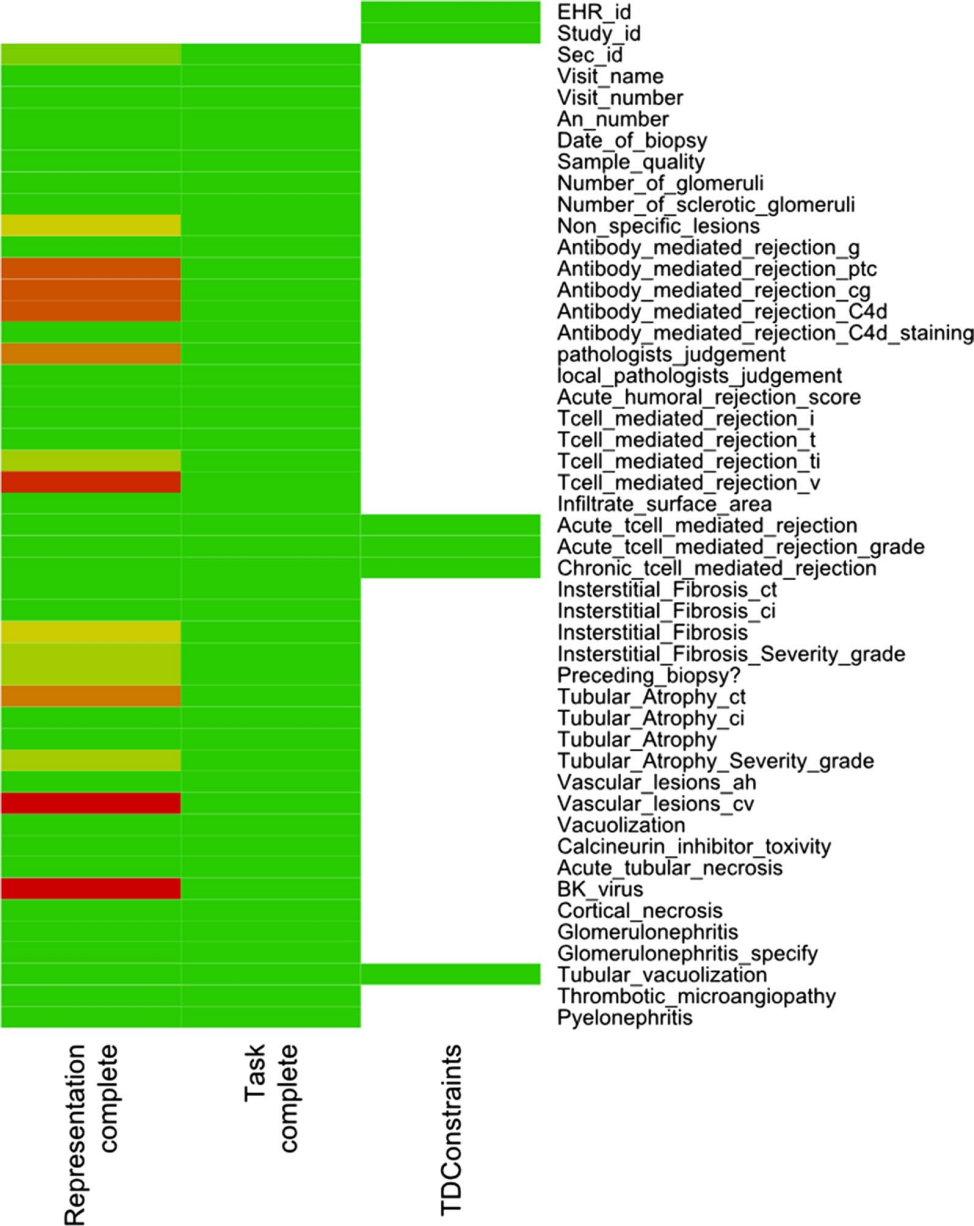
Example heatmap showing DQ-assessment results for measures RepresentationComplete, TaskComplete and TDConstraints per variable

In our study, we derived constraints from the consented templates without adding any more restrictive constraints. Note that the MMs checking CIM-constraints were not hard-coded for this particular assessment, but derived automatically from CIMs. Therefore, our created example knowledge base containing the HDQF-MMs is applicable in any sensible use case and applying the measure TDConstraints on MMs derived from different CIMs is possible without adapting the TDConstraints-MM. This example shows how existing work proposing well-thought-out means for DQ-assessment can be integrated with our approach and demonstrates possibilities for summarizing and assessing MM-results using multi-layered MMs. The HDQF-MMs’ results were summarized in a heatmap (example in Fig. 3). Figure 3 does not include the example MM checking the range for the date of biopsy as depicted in Fig. 2, since the defined range constraint is just an arbitrary example. We mapped the domain paths to shorter variable names for display in Fig. 3.

We executed all MMs using the openCQA client (Fig. 1A). The client resolved dependencies of multi-layered MMs and invoked the MM execution on server-side (Fig. 1B). The server side application extracted the desired input data for each MM (from the dataset or from other MMs’ results), executed the MM in R (Fig. 1C) and returned the results for display on the client side. Finally, we compared openCQA’s MM-results with those of SPSS, to validate correct computation of MM-results in openCQA.

## Results

The dataset of the study comprised 384 variables. On a subset of 65 variables belonging to two templates, the absolute and relative frequencies were of interest. openCQA derived 245 and 381 MMs respectively from reference model types and CIMs. After excluding MMs derived from CIMs and MMs grouping for dimensions, 67 and 115 MMs were left. The automatically derived MMs included the frequencies and percent values of interest, measures like minimum, maximum, median and mean as well as appropriate visualizations as needed for the assessment of the study’s data. Table 5 lists example results. The histograms for the distributions of age and time between transplantation and posttransplant biopsy were not generated automatically, but were added manually using openCQA’s GUI. Since checking the age and gender distributions in datasets is common practice in clinical studies, these MMs are well suited to be part of a task-specific knowledge base (Fig. 1F), e.g. for initial data analysis in studies (cf. [49]). Patient and donor age and gender were expressed conforming to internationally governed archetypes. Thus, such a knowledge base entails MMs creating histograms as characterization (cf. Fig. 2E) with the respective archetype-paths from the international archetypes as domain paths (cf. Fig. 2B) and tags (cf. Fig. 2A) indicating the task “initial data analysis in studies”. Such MMs could be loaded from the knowledge base and applied on other sensible datasets to visualize age or gender distributions without the need for modifications.

**Table 5:**
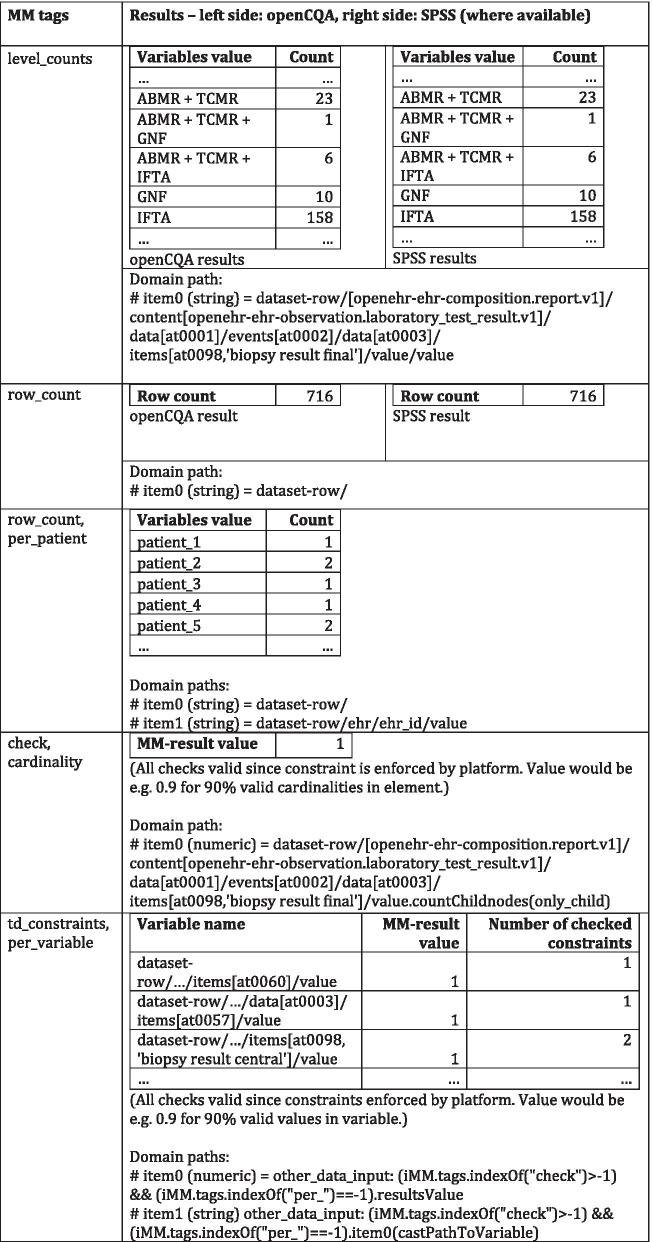
Selected exemplary DQ-assessment results from ROCKET study

MM tags indicate what the MM does (cf. "MM formalization" section). Results from openCQA (left) and matched SPSS results where available (right). Domain paths indicate the variables for which results were calculated

Intention of Table 5 is to illustrate MM-results while not revealing any clinical study results, which are not subject of this work. Frequencies and percent values (relative frequencies) from openCQA’s MM-results and the statistics calculated using SPSS were identical with the exception of one variable where the data integration pipeline did not catch an unexpected null flavor, i.e. source data contained ND instead of NA for some missing values (Error in data integration pipeline, calculations in DQ-assessment were correct). As shown in Table 5, row counts per patient were not calculated in SPSS. The corresponding MM was excluded from openCQA’s MMs before comparing results since it involves grouping in dimensions (cf. "Application of DQ-assessment method" section). Constraint checks (e.g. cardinality check) derived from CIMs and *TDConstraints* from HDQF framework were not available as SPSS-results as well.

The distribution of kidney transplant recipient’s age was not available from SPSS statistics. The other histograms (not shown) for the distributions of age as well as distribution of time in months between last transplantation and the study biopsy were concordant between SPSS and openCQA.

The implemented MMs from the HDQF framework each calculated one MM-result, where each MM-result contained one result-value per variable (cf. Table 5 last row or Fig. 3). Figure 3 shows an example heatmap based on the HDQF measures for one template. *Representation Complete* indicated missing entries for some variables. *Task complete* showed, that no mandatory variable’s values were missing. The measure *TDConstraints* used 12 MMs derived from constraints contained in the corresponding CIM. The assessment part of a heatmap like Fig. 3 is represented using colors. The colors in the heatmap depend on the MM-result values for the respective variable, which serve as input data for the MM plotting the heatmap. Adjusting input values for MMs on certain variables in the heatmap-MM would be an example for more specific DQ-assessment based on task or domain dependent knowledge. For example, Khare et al. (cf. Figure 6 in [8]) showed a similar heatmap where cells with measures for variables not relevant for a study were simply colored white.

## Discussion

We defined two requirements for interoperability in our objectives: portability on standard-compliant technical infrastructure and to base MMs on standardized data definitions. To improve comparability of MM-results and to support collaborative knowledge governance for DQ-assessment, our knowledge-based approach proposes a formalization for DQ-assessment knowledge. We implemented our method for interoperable, knowledge-based DQ-assessment and applied it in the ROCKET study. The generated MMs derived from reference model types and CIM-constraints could serve as basic assessment, e.g. regarding completeness (absolute and relative frequencies) and correctness (constraint checks, frequencies, distribution measures). The presented concepts for formalizing MMs (cf. "MM formalization" section), basing MMs on standardized data definitions (CIMs and archetype-paths), portability (AQL, openEHR REST-API and archetype-paths) and collaborative governance of DQ-assessment knowledge (openEHR CIM governance and compilations of MMs for domains and/or tasks managed using git) worked, produced useful results and showed satisfactory practicability in a real world use case.

### Interoperability

openCQA uses standardized interfaces for data retrieval and our formalized MMs reference variables in datasets using archetype-paths together enabling portability. For example, assuming a hospital in England, which stores its data in an openEHR data repository based on their own templates according to their local application systems (but using international archetypes). This hospital is able to run openCQA and MMs from a German hospital without adaptions as long as MMs base on the international archetypes even if the MMs were created using different templates and an openEHR repository from another vendor.

Using terminologies in CIMs is a supported feature of openEHR, e.g. for describing eligible values. Making use of terminologies would also be useful in DQ-assessment and important for interoperability. Although this task was beyond the scope of the present work, we paid attention that none of our concepts contradicts terminology integration.

Our implementation of the presented concepts for interoperability and knowledge-based DQ-assessment, relies on the openEHR specifications and without adaptions is only applicable to openEHR based data sources. Nevertheless, we took particular care to facilitate expansion of our approach to other data sources and to simplify comparing results between MMs based on different CIM standards. Juarez et al. [26] rely on the ISO/IEC 11179 metadata repository standard. Kapsner et al. [18] state plans to adapt their R-based framework for this standard. Juarez et al. store constraints on valid variable values together with the variable definitions. Their approach is comparable to our proposed CIM-based governance of constraints (Fig. 1E) but remains limited to constraint checks on one variable and does not address the challenge of flexible domain and task specific requirements. For example, such an approach could not implement the row count per patient or the cardinality check shown in Table 5. Comparing MM-results based on constraints defined in a metadata repository and MMs based on openEHR CIMs merely requires mappings between the ISO/IEC 11179 variable definitions and corresponding archetype-paths. Alternative implementations or extensions of openCQA, e.g. to apply our concepts on data sources relying on ISO/IEC 11179 metadata definitions, to the OMOP data model [50] or complying with other CIM-standards like FHIR [51], would increase the value of collaborative knowledge governance. To support that, openCQA is freely available with open source code under MIT License [36]. However, replacing openEHR with other standards or data models affects portability, MM formalization, MM generation and means for knowledge governance. Implications on portability depend on the means other standards provide to support standardized data retrieval, e.g. equivalents of AQL, REST-API and archetype-paths. A fixed common data model like OMOP is sufficient to enable portability of the tool, but of course lacks the benefits of multi-level modelling, e.g. having the same archetype-path for data from different templates. Our proposed MM formalization is usable with other standards/data models just needing another way to reference variables in datasets instead of archetype-paths (again sacrificing the benefits of multi-level modelling). Multi-layered MMs are directly applicable and comparable with openEHR-based MMs (as long as the filter-condition does not address the domain path) since they rely on our MM formalization, not on the openEHR specification. We already approximated application of our concepts to other standards by applying our MMs generated with openCQA (R-scripts) to comparable data in non-openEHR data sources [52]. Implications on MM generation depend on the information contained in the other data models, e.g. datatypes, constraints etc. and on the possibility to govern domain and task specific constraints. openEHR’s means and processes for knowledge-management obviously get lost when using other standards, leaving only git-based knowledge management. Standards like FHIR might be able to substitute this, e.g. by providing other processes and tools [53].

### Knowledge-based DQ-assessment

Considering the possible combinations of variables, checks, groupings and characterizations and keeping in mind that sensible combinations as well as the assessment of MM-results are task and domain dependent, the amount of resultant information could become overwhelming. The finding that selecting sensible MMs and their assessment for a certain task is challenging agrees with findings from the literature, e.g. Diaz-Garelli et al. [21] stress that defining DQ-requirements is complex, resource intensive and typically requires a multidisciplinary team. The intention of our approach is to support DQ-assessment by providing means for flexible generation (MMs from CIMs and from reference model types), reuse and collaborative governance of formalized DQ-assessment knowledge. From the MMs in our use case, we already identified two sensible knowledge bases, i.e. “initial data analysis in studies” and “HDQF”. Diaz-Garelli’s findings support the idea of reusing MMs, since only 17 out of 52 DQ-requirements in their use case were analysis-specific, suggesting good potential for reuse of MMs [21]. Beyond Diaz-Garelli’s approach, we deem most other processes for the elaboration of MMs or proposing MMs for a certain domain or task to be complementary to our approach, not competing, e.g. HDQF [11], 3 × 3 DQA [12] or Henley et al. [54]. Several task-specific implementations for DQ-assessment have been published. These embody valuable task-specific knowledge. In epidemiological research, R-based implementations exist [18, 22, 24, 25]. As Bialke et al. [22] mention, such tools need metadata, e.g. variable definitions, units, code lists etc. to generate specific DQ-reports. This fits well to our CIM and reference model type based MM generation, which inherently provides such metadata. Besides employing such existing R-functions in MMs, our knowledge-based concept can combine them with other MMs and can support the governance of formalized knowledge about sensible tasks for their application and on assessment of their results. Similarly, once implemented as MM-compilation (like the HDQF example), existing DQ-frameworks are enabled to be extended with additional MMs, e.g. for MMs assessing the results of the framework for a certain task. Our method does not limit MMs to certain predefined functions. Even if those new MMs require complex calculations not known yet, tools implementing our concepts do not need to be adapted.

Juarez et al. discuss in which stage of a dataset’s lifecycle DQ-assessment is most worthwhile and focus their framework on this stage [26]. We assume DQ-assessment is sensible at different stages with different perspectives, e.g. a data integration specialist validates data integration locally during implementation, a quality manager continuously monitors DQ in a data integration center and a researcher assesses DQ in a research data network specifically for the research question [8, 15–19]. For this purpose, the presented method is applicable at all stages on a compatible data repository (Fig. 1D) and the concepts we describe support adjusting DQ-assessment to the domain and task.

A common practice to agree on a set of sensible MMs and their assessment for a project is to conduct surveys and reviews with experts and stakeholders [12, 18, 19]. This is similar to openEHR CIM governance which typically involves domain- and technical experts working together to define a CIM’s core data elements, contextual data elements and sensible constraints for a clinical domain (archetypes) or a particular use case (templates). CIM-drafts are refined in multiple review rounds in which experts discuss the draft and suggest improvements finally leading to a consented CIM. Tools to support these review rounds and CIM management over the whole lifecycle are available [32]. We can directly make use of these well-tested processes and tools for CIMs, to govern constraint checks on the data (Fig. 1E). Likewise, for all other types of MMs we can manage knowledge bases (MM-compilations, cf. Fig. 1F) using git [37]. Git primarily supports version control but also comprises features for documentation, discussion and issues tracking that can support similar processes as for CIMs, although less optimized for knowledge governance. Using these two means (CIMs and knowledge bases) for knowledge-management entails the challenge of keeping constraints consistent through updates, which will probably need attention. If we imagine a knowledge base for a certain task that includes MMs derived from a CIM, these MMs are not updated if constraints in the original CIM change and vice versa, the CIM will not change when the respective MMs are adapted. This could be even more complicated if MMs would be aligned with other interoperability standards, e.g. ISO/IEC 11179 metadata repositories [26] or HL7 FHIR [51]. However, we just started collaborative governance of DQ-assessment knowledge and so far did not evaluate different processes regarding their goal to improve task and domain specific DQ-assessments while keeping efforts justifiable. We need more experience in how to combine different means.

## Conclusions

The presented work describes a method for interoperable and knowledge-based DQ-assessment. We provide applicable concepts and a tested exemplary open source implementation. The main contributions our work adds to existing work in the field are to address interoperability (portability and comparability) in DQ-assessment, a knowledge-based approach that considers the need for task and domain specific requirements and flexibility in the types of applicable MMs. Regarding interoperability, we accomplish portability and support MM-comparability through use of standardized interfaces and use of archetype-paths as means to align data from different sources. We demonstrate how MMs generated from openEHR CIMs and reference model types can support DQ-assessment. We propose a formalization for MMs and show means for collaborative governance of DQ-assessment knowledge striving to base DQ-assessment on formalized knowledge. We applied our concepts in a real world use case with satisfactory results, using openCQA as our implementation. Important next steps would be to work on methods for learning of DQ-assessment knowledge, on integrating existing processes for MM elaboration, integrating existing frameworks proposing MMs [11, 12, 21, 54] as well as to gain experience with collaborative governance of DQ-assessment knowledge.

## Supplementary Information

**Additional file 1: Appendix A.** Example MM.

**Additional file 2: Appendix B.** Example AQL.

**Additional file 3: Appendix C.** Adaptions to HDQF-framework’s MMs.

## Data Availability

Restrictions apply to the availability of the patient data, which were used under consent for the ROCKET study, and so are not publicly available. Please contact the corresponding author for data sharing requests. Source code of implemented methods is publicly available at https://gitlab.plri.de/tute/openehr-dq.

## Abbreviations

MM: Measurement method
DQ: Data quality
CIM: Clinical information model
AQL: Archetype query language
SQL: Structured query language

## References

[1] Safran C (2014). Reuse of clinical data. Yearb Med Inform.

[2] Martin-Sanchez FJ, Aguiar-Pulido V, Lopez-Campos GH, Peek N, Sacchi L (2017). Secondary use and analysis of big data collected for patient care. Contribution from the IMIA Working Group on Data Mining and Big Data Analytics. Yearb Med Inform.

[3] Ancker JS, Shih S, Singh MP, Snyder A, Edwards A, Kaushal R (2011). Root causes underlying challenges to secondary use of data. AMIA Annu Symp Proc AMIA Symp.

[4] Botsis T, Hartvigsen G, Chen F, Weng C (2010). Secondary use of EHR: data quality issues and informatics opportunities. AMIA Joint Summits Transl Sci Proc AMIA Joint Summits Transl Sci.

[5] Weiskopf NG, Weng C (2013). Methods and dimensions of electronic health record data quality assessment: enabling reuse for clinical research. J Am Med Inform Assoc.

[6] Huser V, DeFalco FJ, Schuemie M, Ryan PB, Shang N, Velez M (2016). Multisite evaluation of a data quality tool for patient-level clinical data sets. EGEMS (Washington, DC).

[7] Reimer AP, Milinovich A, Madigan EA (2016). Data quality assessment framework to assess electronic medical record data for use in research. Int J Med Inform.

[8] Khare R, Utidjian L, Ruth BJ, Kahn MG, Burrows E, Marsolo K (2017). A longitudinal analysis of data quality in a large pediatric data research network. J Am Med Inform Assoc.

[9] Saez C, Liaw ST, Kimura E, Coorevits P, Garcia-Gomez JM (2019). Guest editorial: special issue in biomedical data quality assessment methods. Comput Methods Programs Biomed.

[10] Liaw ST, Rahimi A, Ray P, Taggart J, Dennis S, de Lusignan S, et al. Towards an ontology for data quality in integrated chronic disease management: a realist review of the literature. Int J Med Inform. 2013;82(1):10–24. 10.1016/j.ijmedinf.2012.10.001. Epub 2012 Nov 2. Erratum in: Int J Med Inform. 2013;82(2):139. PMID: 23122633. 10.1016/j.ijmedinf.2012.10.001 23122633

[11] Johnson SG, Speedie S, Simon G, Kumar V, Westra BL (2016). Application of an ontology for characterizing data quality for a secondary use of EHR data. Appl Clin Inform.

[12] Weiskopf NG, Bakken S, Hripcsak G, Weng C (2017). A data quality assessment guideline for electronic health record data reuse. eGEMs (Gener Evid Methods Improve Patient Outcomes).

[13] Kahn MG, Brown JS, Chun AT, Davidson BN, Meeker D, Ryan PB (2015). Transparent reporting of data quality in distributed data networks. eGEMs (Gener Evid Methods Improve Patient Outcomes).

[14] Johnson SG, Speedie S, Simon G, Kumar V, Westra BL (2015). A data quality ontology for the secondary use of EHR data. AMIA Annu Symp Proc AMIA Symp.

[15] Walker KL, Kirillova O, Gillespie SE, Hsiao D, Pishchalenko V, Pai AK (2014). Using the CER Hub to ensure data quality in a multi-institution smoking cessation study. J Am Med Inform Assoc.

[16] Priest EL, Klekar C, Cantu G, Berryman C, Garinger G, Hall L (2014). Developing electronic data methods infrastructure to participate in collaborative research networks. eGEMs (Gener Evid Methods Improve Patient Outcomes).

[17] Welch G, Recklinghausen FV, Taenzer A, Savitz L, Weiss L (2017). Data cleaning in the evaluation of a multi-site intervention project. eGEMs (Gener Evid Methods Improve Patient Outcomes).

[18] Kapsner LA, Kampf MO, Seuchter SA, Kamdje-Wabo G, Gradinger T, Ganslandt T (2019). Moving towards an EHR data quality framework: the MIRACUM approach. Stud Health Technol Inform.

[19] Khare R, Utidjian LH, Razzaghi H, Soucek V, Burrows E, Eckrich D (2019). Design and refinement of a data quality assessment workflow for a large pediatric research network. EGEMS (Wash DC).

[20] Stausberg J, Bauer U, Nasseh D, Pritzkuleit R, Schmidt CO, Schrader T (2019). Indicators of data quality: review and requirements from the perspective of networked medical research. GMS Medizinische Informatik Biometrie und Epidemiologie.

[21] Diaz-Garelli JF, Bernstam EV, Lee M, Hwang KO, Rahbar MH, Johnson TR (2019). DataGauge: a practical process for systematically designing and implementing quality assessments of repurposed clinical data. EGEMS (Wash DC).

[22] Bialke M, Rau H, Schwaneberg T, Walk R, Bahls T, Hoffmann W (2017). mosaicQA—a general approach to facilitate basic data quality assurance for epidemiological research. Methods Inf Med.

[23] Estiri H, Stephens K (2017). DQe-v: a database-agnostic framework for exploring variability in electronic health record data across time and site location. eGEMs.

[24] MOQA|toolpool Gesundheitsforschung [Internet]. Berlin: Technologie- und Methodenplattform für die vernetzte medizinische Forschung e.V.; c2020. https://www.toolpool-gesundheitsforschung.de/produkte/moqa. Accessed 28 Feb 2020.

[25] LibreUMG/dataquieR [Internet]. https://gitlab.com/libreumg/dataquier. Accessed 9 Sep 2020.

[26] Juárez D, Schmidt E, Stahl-Toyota S, Ückert F, Lablans M (2019). A generic method and implementation to evaluate and improve data quality in distributed research networks. Methods Inf Med.

[27] Johnson SG, Pruinelli L, Hoff A, Kumar V, Simon GJ, Steinbach M (2019). A framework for visualizing data quality for predictive models and clinical quality measures. AMIA Joint Summits Transl Sci Proc AMIA Joint Summits Transl Sci.

[28] Gehring S, Eulenfeld R (2018). German medical informatics initiative: unlocking data for research and health care. Methods Inf Med.

[29] Semler SC, Wissing F, Heyder R (2018). German medical informatics initiative. Methods Inf Med.

[30] Haarbrandt B, Schreiweis B, Rey S, Sax U, Scheithauer S, Rienhoff O (2018). HiGHmed—an open platform approach to enhance care and research across institutional boundaries. Methods Inf Med.

[31] Welcome to openEHR [Internet]. London: openEHR Foundation; c2017. http://www.openehr.org/. Accessed 2 Nov 2017.

[32] Clinical Knowledge Manager [Internet]. London: openEHR Foundation; c2020. https://www.openehr.org/ckm. Accessed 20 Jan 2021.

[33] Wulff A, Haarbrandt B, Marschollek M (2018). Clinical knowledge governance framework for nationwide data infrastructure projects. Stud Health Technol Inform.

[34] Wulff A, Sommer KK, Ballout S, Haarbrandt B, Gietzelt M, HiGHmed Consortium (2019). A report on archetype modelling in a nationwide data infrastructure project. Stud Health Technol Inform.

[35] Archetype Query Language (AQL) [Internet]. London: openEHR Foundation; c2020. https://specifications.openehr.org/releases/QUERY/latest/AQL.html. Accessed 20 Feb 2020.

[36] Erik Tute/openCQA · GitLab [Internet]. Braunschweig: Peter L. Reichertz Institut für Medizinische Informatik der Technischen Universität Braunschweig und der Medizinischen Hochschule Hannover; c2020. https://gitlab.plri.de/tute/openehr-dq. Accessed 20 Feb 2020.

[37] Git [Internet]. Git community. https://git-scm.com/site. Accessed 4 Jun 2020.

[38] Drools - Drools - Business Rules Management System (Java™, Open Source) [Internet]. Raleigh: Red Hat, Inc.; c2006–2017. http://www.drools.org/. Accessed 2 Nov 2017.

[39] Health Level Seven International - Homepage [Internet]. Ann Arbor: Health Level Seven International; c2007–2017. http://www.hl7.org/Special/Committees/arden/index.cfm. Accessed 2 No 2017.

[40] About the Object Constraint Language [Internet]. Needham: Object Management Group; c2020. https://www.omg.org/spec/OCL/. Accessed 21 Feb 2020.

[41] Guidline Definition Language (GDL) [Internet]. London: openEHR Foundation; c2020. https://specifications.openehr.org/releases/CDS/latest/GDL.html. Accessed 21 Feb 2020.

[42] Reclassification using OmiCs integration in KidnEy Transplantation (ROCKET)—ERA-LEARN [Internet]. Berlin: VDI/VDE Innovation + Technik GmbH. https://www.era-learn.eu/network-information/networks/eracosysmed/2nd-joint-transnational-call-for-european-research-projects-on-systems-medicine/reclassification-using-omics-integration-in-kidney-transplantation-rocket. Accessed 1 Apr 2020.

[43] Platform | Better care [Internet]. Ljubljana: Better d.o.o.; c2019. https://www.better.care/. Accessed 27 Aug 2020.

[44] Brown JS, Kahn M, Toh D (2013). Data quality assessment for comparative effectiveness research in distributed data networks. Med Care.

[45] Venet D, Doffagne E, Burzykowski T, Beckers F, Tellier Y, Genevois-Marlin E (2012). A statistical approach to central monitoring of data quality in clinical trials. Clin Trials.

[46] Sunderland KM, Derek B, Fraser J, Kwan D, McLaughlin PM, Montero-Odasso M (2019). The utility of multivariate outlier detection techniques for data quality evaluation in large studies: an application within the ONDRI project. BMC Med Res Methodol.

[47] Tute E, Wulff A, Marschollek M, Gietzelt M (2019). Clinical information model based data quality checks: theory and example. Stud Health Technol Inform.

[48] Richter A, Schössow J, Werner A, Schauer B, Radke D, Henke J (2019). Data quality monitoring in clinical and observational epidemiologic studies: the role of metadata and process information. GMS Medizinische Informatik Biometrie und Epidemiologie.

[49] Huebner M, Le Cessie S, Schmidt C, Vach W (2018). A contemporary conceptual framework for initial data analysis. Obs Stud.

[50] OMOP Common Data Model—OHDSI [Internet]. Observational Health Data Sciences and Informatics; c2020 [cited 21 Feb 20]. https://www.ohdsi.org/data-standardization/the-common-data-model/. Accessed 21 Feb 2020.

[51] Index - FHIR v.4.0.1 [Internet]. Ann Arbor: Health Level Seven International; c2020. https://www.hl7.org/fhir/. Accessed 2 Mar 2020.

[52] Kindermann A, Tute E, Benda S, Löpprich M, Richter-Pechanski P, Dietrich C. Preliminary analysis of structured reporting in the HiGHmed use case cardiology: challenges and measures. Stud Health Technol Inform (Forthcoming). 10.3233/SHTI210068 34042893

[53] The FHIR collaborative platform - SIMPLIFIER.NET [Internet]. Firely; c2020. https://simplifier.net/. Accessed 20 Jan 2021.

[54] Henley-Smith S, Boyle D, Gray K (2019). Improving a secondary use health data warehouse: proposing a multi-level data quality framework. EGEMS (Wash DC).

