# Supplementary material for: A method for interoperable knowledge-based data quality assessment
Source: BMC Med Inform Decis Mak. 2021 Mar 9;21:93. doi: 10.1186/s12911-021-01458-1 (PMC7942002; doi:10.1186/s12911-021-01458-1)
Supplement: Supplementary file 3 — Additional file 3: Appendix C. Adaptions to HDQF-framework’s MMs. [file 12911_2021_1458_MOESM3_ESM.docx]

| **MM-name HDQF** | **MM-name adapted** | **Comment** |
| --- | --- | --- |
| Representation Complete | Representation Complete | No change |
| DomainComplete | Task complete | In HDQF the domain, i.e. the clinical content of the variable, defines if the variable is mandatory. In our case, the task defined which variables were mandatory, thus we renamed it. |
| DomainConstaints | TDConstraints | Johnson et al. [14] propose a domain and a task ontology to account for the domain and task dependency of data quality and mention openEHR CIMs as possibility for domain ontologies. However, CIMs can serve to express both domain and task-dependent constraints. Accordingly, we renamed the MM and implemented *TDConstraints* to support multiple CIMs for the same clinical concept, i.e. for domain and for task dependent constraints. |

Appendix C - Adaptions to HDQF-framework’s MMs
